# Feasibility of a novel wearable thermal device for management of bothersome hot flashes in patients with prostate cancer

**DOI:** 10.1038/s41391-023-00771-2

**Published:** 2023-12-13

**Authors:** Pamela Peeke, Sonja K. Billes, Andrew Vetter, Nader Naghavi, Diana Le, Matthew Smith, Alicia K. Morgans

**Affiliations:** 1 Embr Labs, Inc, Boston, MA USA; 2 Kelly Statistical Consulting, Carlsbad, CA USA; 3 Dana-Farber Cancer Institute, Boston, MA USA

**Keywords:** Prostate cancer, Prostate cancer

## Abstract

**Background:**

This single-arm prospective study evaluated the feasibility of a novel wrist-worn thermal device that applies cooling to the inside of the wrist for management of bothersome hot flashes in prostate cancer survivors.

**Methods:**

57 individuals were enrolled and instructed to use the thermal device as needed for management of hot flashes for 4 weeks. The primary outcome was thermal device usage (hours and sessions per day). Additional outcomes included the change in Hot Flash Related Daily Interference Scale (HFRDIS, range 0–10) and Patient Reported Outcomes Measurement Information System Sleep Disturbance 4a (PROMIS SD T-score, range 0–100) and Sleep-Related Impairment 8a (PROMIS SRI T-score, range 0–100). Study procedures were conducted remotely from May to Dec 2021 in the US.

**Results:**

44 participants completed the study and 39 had retrievable usage data. The mean ± SD age was 67 ± 6 years and 5 ± 5 years since cancer diagnosis. The baseline mean ± SD HFRDIS score of 4.3 ± 2.0 indicated moderate hot flash interference in this population. During the study, participants used the thermal device (mean ± SD) 3.2 ± 2.5 hours/day and 7.6 ± 3.6 sessions/day. Most (67%) participants reported using the device 7 days and 7 nights each week. Statistically significant improvements from baseline at Week 4 were observed for HFRDIS (mean ± SE change: −1.1 ± 0.3), PROMIS SD (−6.0 ± 1.0), and PROMIS SRI (−5.5 ± 1.2) scores (all *p* < 0.001). The majority (69%) of participants reported that the thermal device was effective at helping them manage hot flashes. No adverse events were reported.

**Conclusions:**

Results support the feasibility of using the thermal device for management of bothersome hot flashes in prostate cancer survivors. Future randomized controlled studies are warranted to evaluate the impact of the thermal device on frequency and severity of hot flashes, sleep quality, fatigue, and overall quality of life.

## Introduction

Prostate cancer affects approximately 1 in 8 men and is the most common cancer in men in the US [1]. Androgen deprivation therapy (ADT) is the primary systemic therapy used to treat prostate cancer, and up to half of patients are exposed to ADT during the course of their treatment [2, 3]. Although ADT is highly effective for mitigating prostate cancer growth, it is associated with numerous negative side effects, including hot flashes [4–7].

Hot flashes and night sweats are vasomotor symptoms that are characterized by a sudden sensation of intense heat, usually accompanied by cutaneous vasodilation and sweating [5, 6]. Hot flashes can be accompanied by heart palpitations and feelings of anxiety or panic and are sometimes followed by chills. Hot flashes have been reported by 80% or more of prostate cancer patients undergoing ADT, and hot flashes may continue even after stopping ADT, particularly among patients without testosterone recovery despite treatment cessation [5, 8, 9]. Hot flashes resulting from ADT contribute to physical and mental distress [10] and are associated with increased sleep disturbance, diminished cognitive function, and lower quality of life [4, 6, 11, 12]. Approximately 27% of prostate cancer patients report that hot flashes are the most troublesome side effect of ADT, and some individuals discontinue ADT due to severe and debilitating hot flashes [5]. Despite the negative impact of hot flashes, current strategies for treatment in prostate cancer patients are limited by variable efficacy and unacceptable side effects [5, 7].

There is an unmet need for effective and well-tolerated hot flash management strategies that improve both prostate cancer survivorship and the subjective patient experience [13]. Digital technology offers a novel opportunity to address gaps in symptom management [14]. A novel wrist-worn thermal device (Embr Wave®, Embr Labs, Boston, MA) applies low-intensity dynamic cooling to the inside of the wrist and may aid in management of hot flashes in prostate cancer survivors [15]. In peri- and post-menopausal women with bothersome hot flashes, use of the thermal device improved subjective measures of hot flash interference, thermal comfort, and hot flash control [16]. This prospective, single-arm, study evaluated the feasibility of the thermal device for management of bothersome hot flashes in prostate cancer survivors.

## Materials and methods

### Study design and participants

This prospective, single-arm, feasibility study was conducted in the United States between May and December 2021. Participants were recruited via social media and from prostate cancer support groups (ZERO, AnCan, UsTOO). Study procedures were conducted remotely. All patient reported outcomes were collected electronically via Research Electronic Data Capture (REDCap Cloud, Encinitas, CA), a remote, web-based, secure, HIPAA-compliant, data collection platform. Study support was provided to participants by phone calls and texts with study staff. Participants had the option for a telephone consultation with the study staff at any point during the study. Prior to study initiation, the study protocol was reviewed and approved by The Argus Institutional Review Board (Argus IRB, Tucson, AZ). The study is registered at clinicaltrials.gov (NCT04892914). The study was performed in accordance with the Declaration of Helsinki. Informed consent was obtained from all participants prior to enrolling in the study.

Interested individuals accessed a REDCap self-report screening survey by following a weblink from the study brochure. To be eligible, participants had a history of prostate cancer treatment (ADT, hormone therapy, or orchiectomy) and experienced bothersome hot flashes (defined as at least 28 hot flashes per week and at least moderately bothersome) for at least 30 days. Eligible participants also reported having a working smartphone (iPhone 6 or higher, Android 8.0 or higher). Participants were ineligible if they reported any planned new prostate cancer medical treatment or treatment changes during the study, had a history of a sleeping disorder other than insomnia, were undergoing treatment for insomnia, were taking prescription sleep medications, were taking prescription or over-the-counter medications known to modify hot flashes, reported regular use of alcohol (more than 7 drinks per week), or reported any other significant medical or psychiatric illness. Participants successfully completed the study if they completed all electronic assessments. Prior to starting the study, participants were informed that they could keep the thermal device upon completing the study.

### Intervention

The thermal device (Embr Wave, Embr Labs, Boston, MA) utilizes a thermoelectric (Peltier) heat pump to modulate temperature against the wearer’s inner wrist and provides either cooling or heating upon activation [15, 16]. Buttons on the device allow the user to start or stop a session and adjust the temperature level, with additional settings available on the companion mobile app. The thermal device automatically logs use over time and is charged using a microUSB cable.

During the 4-week study, participants were instructed to use the thermal device as needed during the day and night for management of hot flashes. Technical onboarding occurred 1 to 3 days prior to study start so participants could familiarize themselves with the device. Participants were mailed the thermal device and provided with a training video that showed how to set up and use the thermal device. Participants were instructed to sync the thermal device with their mobile phone daily and to charge it daily to ensure no loss of function.

### Outcomes and assessments

The primary outcome was use of the thermal device (number of hours and sessions per day). Ad hoc analyses were conducted to evaluate associations between thermal device use and participant demographics (eg, age, time since cancer diagnosis), hormone therapy medication, and baseline assessment scores (eg, HFRDIS, PROMIS SRI, PROMIS SD).

Secondary outcomes included validated patient-reported outcome measures (PROMs). Hot flash interference was assessed weekly using the Hot Flash Related Daily Interference Scale (HFRDIS), a validated, reliable, 10-item self-report measure that assesses the impact of hot flashes on quality of life in midlife women [17]. A minimal important difference (MID) of 1.66 has been established in midlife women [18].

Sleep was evaluated at Weeks 2 and 4 using the following validated, reliable assessments: Patient-Reported Outcomes Measurement Information System Sleep Disturbance 4a (PROMIS SD; v1.0, 2016), PROMIS Sleep-Related Impairment 8a (PROMIS SRI; v1.0, 2016) [19, 20], and the Epworth Sleepiness Scale (ESS) [21]. PROMIS measures are a set of health-related measures created under the National Institute of Health (NIH) to improve patient outcomes. PROMIS SD assesses quality, depth, and restoration related to sleep (eg, “My sleep was refreshing”), and the PROMIS SRI assesses alertness, sleepiness, and tiredness while awake (eg, “I felt irritable because of poor sleep”) over the previous 7 days. PROMIS SD and PROMIS SRI total scores are represented as a T-score metric where 50 is the mean of a general US adult reference population and 10 is the standard deviation (SD) of that reference population. The ESS is a widely used measure of daytime sleepiness where participants are asked about their level of sleepiness in the previous 2 weeks.

Additional unvalidated exploratory surveys assessed experience with daytime hot flashes (during the daytime while awake or out of bed) and nighttime hot flashes and night sweats (during the nighttime or while sleeping) at baseline and each week of the study. Participants were asked to report the subjective number and duration of hot flashes and night sweats, level of bother, perceived control over hot flashes and night sweats, and interference of hot flashes and night sweats with daytime activities or sleep. Any adverse effects were reported retrospectively by study participants in weekly surveys. An electronic exit survey at the end of the study evaluated self-report user acceptance and use of the device.

### Statistical analysis

The primary outcome was calculated by summarizing thermal device usage data (automatically logged by the device and synced to the cloud backend through the mobile phone). Continuous variables were summarized using descriptive statistics and categorical variables were summarized as number or percentage of participants. Changes from baseline in PROMs were analyzed using paired 2-tailed t-tests. Because this was an exploratory pilot study, no adjustments were made for the multiple tests performed. Analyses were performed using SAS for Windows statistical software, version 9.4 or higher (SAS, Cary, NC), except where other software was deemed more appropriate. Spearman’s rank order correlations and Mann–Whitney U tests were used to evaluate associations between thermal device use and participant characteristics or baseline assessment scores. A *p*-value of *p* < 0.05 was considered statistically significant.

## Results

### Study population and baseline characteristics

**Fig. 1:**
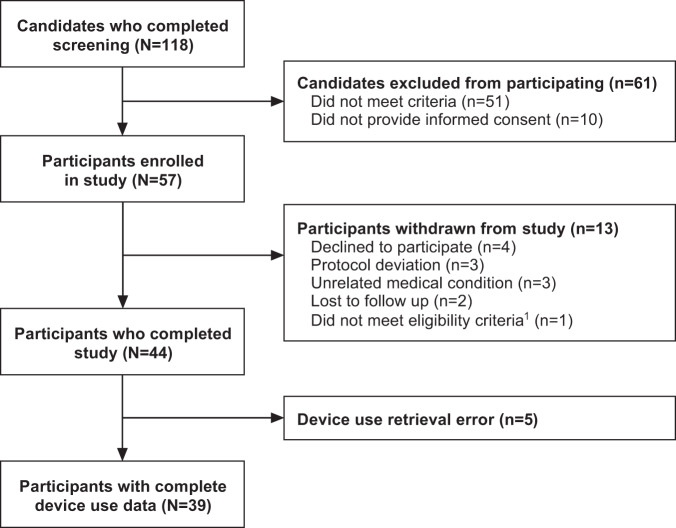
Participant flow during the study. ^1^ Participant was taking prohibited medication.

Of the 118 individuals screened, 57 were eligible and enrolled in the study (Fig. 1). Of these, 44 completed the 4-week intervention and 39 had retrievable usage data (thermal device use was unretrievable for 5 participants). The mean participant age was 67 years and time since prostate cancer diagnosis was 5 years; 36 participants reported taking hormone therapy medication (Table 1).

**Table 1:** Demographics and baseline characteristics.

Parameter	Mean ± SD or *n*
Age, years	67.3 ± 6.2 (range: 57 to 78)
Time since diagnosis, years	4.9 ± 5.0 (range: 1 to 23)
Hot flash bothersome rating (range: 0–10)	7.8 ± 1.6
BMI, kg/m^2^	27.1 ± 3.9
Race and ethnicity	
White	39
Black	3
Asian	1
Hispanic or Latino	1
Hormone therapy	
None reported	8
Any	36
Leuprorelin	22
Abiraterone acetate	13
Enzalutamide	6
Degarelix	2
Bicalutamide	2
Relugolix	2
Apalutamide	1

Data are for the Completer Population (*N* = 44). Demographics and baseline characteristics were self-report. Participants could report taking more than one hormone therapy medication.

### Safety and tolerability

No AEs or tolerability issues were reported during the study.

### Use of the thermal device

**Fig. 2:**
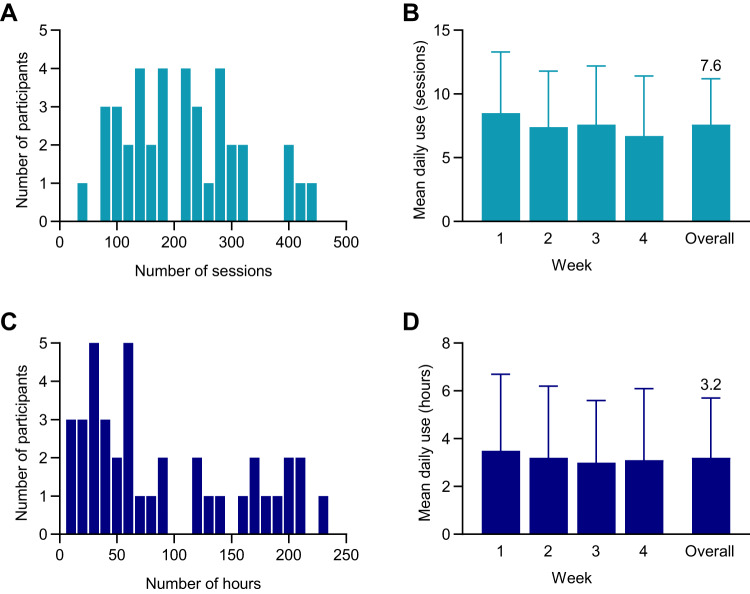
Use of the thermal device. Total number of sessions (**A**) or hours (**C**) per participant for the 4-week intervention. Daily use (mean and SD) per participant in sessions (**B**) and hours (**D**). Data are shown for participants with retrievable usage data (*N* = 39).

Among participants who completed the study and who had retrievable usage data (*N* = 39), daily mean ± SD use of the thermal device over the 4-week study was 7.6 ± 3.6 sessions (median: 7.5, IQR: 4.8–10.3) sessions totaling 3.2 ± 2.5 h (median: 2.3, IQR: 1.1–5.6) per participant (Fig. 2). Total use of the thermal device over the 4-week study varied among participants, although mean daily use was similar each study week (Fig. 2). Although the device produces either warming or cooling sessions, participants exclusively used cooling.

No statistically significant associations were observed between thermal device use and demographics (e.g., age, time since cancer diagnosis), hormone therapy medication, or baseline assessment scores.

### Hot flash interference

**Fig. 3:**
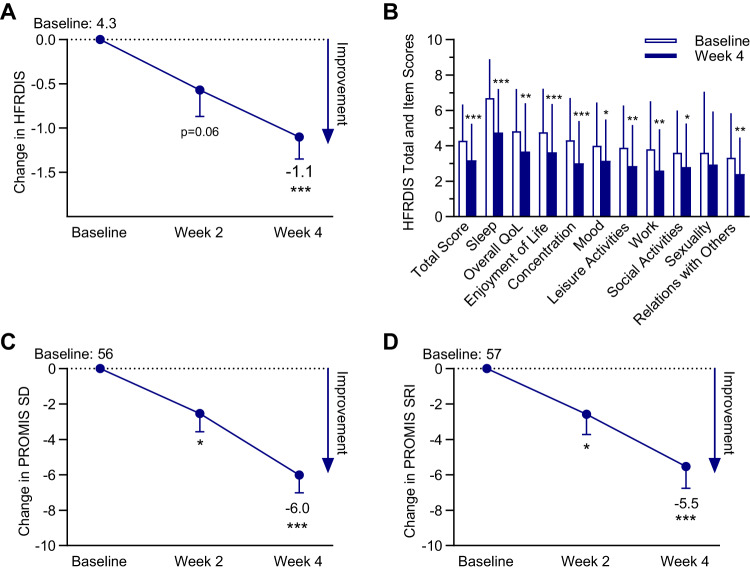
Measures of hot flash interference and sleep. Data are mean and SE change from baseline (**A**, **C**, **D**) or mean and SD (**B**) for the completer population (*N* = 44). *p < 0.05, **p < 0.01, ***p < 0.001 vs baseline. HFRDIS Total Score is the cumulative sum of responses divided by the number of items; Range: 0 to 10. Higher score indicates greater hot flash interference. HFRDIS=hot flash related daily interference scale. PROMIS Sleep Disturbance 4a and PROMIS Sleep-Related Impairment 8a scores are represented as a T score, which is normalized and calibrated against the US population (United States population average score, 50; 10 points = 1 SD). *PROMIS* Patient-Reported Outcomes Measurement Information System.

The mean ± SD baseline HFRDIS total score was 4.3 ± 2.0 (Table 2, Fig. 3), indicating moderate hot flash interference [18]. HFRDIS total score decreased throughout the study, with a decrease of −1.1 ± 0.3 (*p* < 0.001) observed at Week 4 (Table 2, Fig. 3). Statistically significant (*p* < 0.05) reductions from baseline to Week 4 were observed in the HFRDIS items of hot flash interference with: sleep, overall quality of life, enjoyment of life, concentration, mood, leisure activities, work, social activities, and relations with others (Table 2).

**Table 2:** Participant assessments.

	Baseline (mean ± SD)	Week 4 (mean ± SD)	Change from baseline at Week 4 (mean ± SE)	*P*-value for change
HFRDIS total score	4.3 ± 2.0	3.2 ± 2.0	−1.1 ± 0.3	<0.001
Work	3.8 ± 2.7	2.6 ± 2.3	−1.2 ± 0.4	0.004
Social activities	3.6 ± 2.4	2.8 ± 2.5	−0.8 ± 0.3	0.022
Leisure activities	3.9 ± 2.4	2.9 ± 2.3	−1.0 ± 0.4	0.007
Sleep	6.7 ± 2.2	4.8 ± 2.5	−2.0 ± 0.4	<0.001
Mood	4.0 ± 2.4	3.2 ± 2.4	−0.8 ± 0.3	0.018
Concentration	4.3 ± 2.4	3.0 ± 2.4	−1.3 ± 0.4	<0.001
Relations with others	3.3 ± 2.5	2.4 ± 2.0	−0.9 ± 0.3	0.007
Sexuality	3.6 ± 3.4	3.0 ± 3.0	−0.7 ± 0.4	0.129
Enjoyment of life	4.8 ± 2.5	3.6 ± 2.7	−1.1 ± 0.3	<0.001
Overall quality of life	4.8 ± 2.4	3.7 ± 2.7	−1.1 ± 0.4	0.003
PROMIS SD T-score	56.4 ± 6.7	50.4 ± 7.9	−6.0 ± 1.0	<0.001
PROMIS SRI T-score	56.8 ± 9.5	51.3 ± 9.8	−5.5 ± 1.2	<0.001
ESS score	7.2 ± 3.5	6.4 ± 3.6	−0.8 ± 0.4	0.058
Daytime hot flashes				
Number	6.6 ± 2.6	5.0 ± 2.7	−1.6 ± 0.3	<0.001
Bothersome rating	5.7 ± 2.8	3.7 ± 2.4	−2.0 ± 0.4	<0.001
Interference with activities	4.2 ± 2.6	3.2 ± 2.5	−1.0 ± 0.4	0.009
Control over interference	2.5 ± 2.4	4.0 ± 2.9	1.5 ± 0.5	0.006
Nighttime hot flashes/night sweats				
Number	4.0 ± 2.4	3.4 ± 2.4	−0.6 ± 0.4	0.09
Bothersome rating	5.9 ± 3.1	3.9 ± 2.5	−2.1 ± 0.4	<0.001
Interference with sleep	6.4 ± 3.1	4.0 ± 2.5	−2.4 ± 0.5	<0.001
Control over interference	1.6 ± 1.7	3.5 ± 2.9	1.9 ± 0.5	0.001

Data are for the completer population (*N* = 44). HFRDIS Total Score is the cumulative sum of responses divided by the number of items; Range: 0–10. Higher score indicates greater hot flash interference. PROMIS SD and PROMIS SRI scores are represented as a T score, which is normalized and calibrated against the US population (United States population average score, 50; 10 points = 1 standard deviation [SD]). Subjective ratings of daytime hot flashes and nighttime hot flashes/night sweats were assessed retrospectively over the previous week on a scale from 1 to 10. Hot flash number responses were from 0 to 10 or >10. Hot flash bothersome rating responses range: 0 (not at all) to 10 (extremely). Hot flash interference response range: 0 (not at all) to 10 (extremely). Hot flash control response range: 0 (no control) to 10 (total control).

*ESS* Epworth Sleepiness Scale, *HFRDIS* hot flash related daily interference scale, *PROMIS* Patient-Reported Outcomes Measurement Information System.

### Sleep disturbance and daytime fatigue

The mean ± SD baseline PROMIS SD and SRI T-scores were 56 ± 7 and 57 ± 10, respectively, indicating mild impairment (Table 2, Fig. 3). Statistically significant improvements were observed from baseline to Week 4 in sleep disturbance and sleep-related impairment. Daytime sleepiness was within the normal range at baseline and no statistically significant change in score was observed (Table 2).

### Subjective hot flash ratings

At baseline, participants reported experiencing a mean ± SD of 6.6 ± 2.6 hot flashes during the daytime (while awake or out of bed) and 23% (*N* = 10/44) reported having 10 or more hot flashes/day. The majority of participants (*N* = 23/44, 52%) reported that daytime hot flashes lasted between 1–3 min (answer options: less than 1 min, 1–3 min, 4–5 min, greater than 5 min). Participants reported experiencing a mean of 4.0 ± 2.4 nighttime hot flashes (while in bed or asleep). Most participants (*N* = 31/44, 70%) reported being bothered by nighttime hot flashes, while 59% (*N* = 26/44) reported being bothered by daytime hot flashes.

Statistically significant differences from baseline to Week 4 were observed in subjective ratings of hot flash number, hot flash bother, hot flash interference with daily life or sleep, and control over interference from hot flashes (Table 2).

### Self-report use of the device and device satisfaction

Among individuals who completed the study, the majority (*N* = 29/44, 66%) reported using the thermal device 7 days/week and 7 nights/week. Additionally, 66% (*N* = 29/44) rated the device as somewhat to extremely effective at helping them manage both daytime and nighttime hot flashes. Most (*N* = 34/44, 77%) reported being somewhat to very satisfied with the device.

## Discussion

This is the first study to evaluate the feasibility of a personalized wearable thermal device for management of bothersome hot flashes in prostate cancer patients. The thermal device evaluated in this study delivers low intensity dynamic cooling or warming to the inside of the wrist when activated by the user [15], however, participants in this study all elected to use only cooling. Over the course of the 4-week study, average daily use of the thermal device per participant was approximately 8 sessions totaling 3 h. Most participants reported using the device during the day and night for management hot flashes and night sweats. Use of the thermal device (sessions and hours) was similar each week of the study, suggesting that participants felt that wearing and using the device was acceptable for the duration of the 4-week study.

Although originally developed to assess the impact of hot flashes in women, the HFRDIS has been used in studies of men with prostate cancer [22, 23]. We selected the HFRDIS because it includes daily life activities that are affected by hot flashes [17]. Furthermore, in menopausal women, changes in hot flash interference (HFRDIS score) were shown to be significantly correlated with changes in other hot flash variables (e.g., hot flash frequency, severity, and bother) [24]. Participants with prostate cancer who completed this 4-week study reported improvements in hot flash interference (HFRDIS Total Score) and in multiple HFRDIS item scores, including sleep, quality of life, enjoyment of life, and concentration [17]. The change in HFRDIS total score was 1.1, while in midlife women the MID for HFRDIS total is 1.7 [18]. Additionally, improvements in other hot flash-related measures (e.g., subjective ratings of hot flash frequency, interference with daily life/sleep, bother, and control) are consistent with studies of menopausal women [18].

Sleep disturbance is prevalent among prostate cancer survivors, with 50% reporting poor sleep quality [25]. Furthermore, hot flashes are a major contributor to increased sleep disturbance in individuals undergoing ADT [12, 25, 26]. Participants in the current study reported moderate hot flash interference with sleep at baseline in the HFRDIS, while 70% reported being bothered by hot flashes and night sweats at night. Similarly, baseline measures of sleep disturbance and sleep-related impairment (assessed with the PROMIS SD and PROMIS SRI) were slightly elevated. The mean baseline PROMIS SD T-score of 56 is higher than the US PROMIS SD Cancer reference value for prostate cancer of 48 [26] and in other studies of the PROMIS SD in individuals with prostate cancer [27, 28], including a study of the same PROMIS SD 4-item short form evaluated by telephone interviews in men in North Carolina over 12 months (PROMIS SD T-score = 50) [29]. Thus, our study population likely reflects a subpopulation of prostate cancer patients with greater sleep disturbance. Furthermore, the improvements in PROMIS SD and PROMIS SRI observed at Week 4 are consistent with improvements in nighttime hot flashes and night sweats. Conversely, ESS score was within normal at baseline and was unchanged during the study. Additional research to standardize sleep measures and establish clinically meaningful changes is needed in this population [30], and subsequent studies of the thermal device would benefit from inclusion of objective sleep measures.

Most participants (66%) reported using the thermal device during the daytime and nighttime throughout study. Use of the thermal device could reflect adherence to the protocol (eg, using the device as needed for hot flashes), however, 66% of participants also rated the thermal device as effective for management of daytime and nighttime hot flashes, while 77% of participants reported being somewhat to very satisfied with the device. These reports indicate that most participants experienced a benefit or at least found the thermal device acceptable to wear.

The improvements in measures of sleep and hot flashes, as well as device satisfaction, are consistent with findings from a controlled crossover pilot study that evaluated the thermal device in midlife women experiencing insomnia and menopausal hot flashes [16]. Use of the thermal device for 2 weeks was associated with improvements in sleep onset latency, amount of nighttime sleep, insomnia, sleep disturbance, and sleep-related impairment. Participants also reported improved hot flash control and reduced hot flash interference, suggesting a benefit across populations.

Prostate cancer survivorship is complicated by treatment toxicities, age-related comorbidities, and polypharmacy [31, 32]. Providing patient-directed and individualized symptom management is essential to mitigate declines in quality of life [13, 31, 33]. It should be acknowledged that, in addition to hot flashes, there are multiple complications of ADT that affect quality of life and increase risk of comorbidities, including cardiometabolic effects, loss of bone mineral density, sexual dysfunction, fatigue, cognitive decline, and others. Measures to address all of these are necessary to improve the patient experience and overall health during and after treatment for prostate cancer [13, 31]. Evidence suggests that regular exercise has a beneficial effect on fatigue, fitness, physical and cognitive function and also improves quality of life [31, 32, 34, 35]. Because hot flashes are associated with reduced quality of life [5, 36, 37], hot flash management in affected individuals is also a critical component of prostate cancer survivorship. Supportive hot flash management, such as using fans or dressing in layers remains insufficient for many patients, and although estrogen treatment may reduce the incidence of hot flashes and night sweats, estrogen-related side effects (e.g., gynecomastia, increased risk of thrombosis) limit the uptake of this approach [38]. Use of a thermal device may provide a nonpharmacological management option for bothersome hot flashes due to the minimal side effect burden.

Study limitations include the small sample size, 4-week duration, and lack of a control group. The 4-week duration is not without precedent [39, 40], and other studies of hot flashes of a longer duration (usually 8–12 weeks) have demonstrated a plateau effect of the intervention beyond 4 weeks [41, 42]. Further studies of the thermal device for hot flash management would benefit from a randomized controlled design and larger number of participants.

Results of this study support the feasibility of use of the thermal device for management of bothersome hot flashes in prostate cancer survivors. Future randomized controlled studies are warranted to evaluate the impact of the thermal device on the frequency and severity of hot flashes, sleep quality, fatigue, and overall quality of life, in addition to defining the potential utility of the wearable thermal device in prostate cancer survivors experiencing bothersome hot flashes.

## Data Availability

Data used for analysis are available from the corresponding author upon reasonable request.
